# Increased access to *TP53* analysis through breast cancer multi-gene panels: clinical considerations

**DOI:** 10.1007/s10689-017-0020-z

**Published:** 2017-07-05

**Authors:** Jacopo Azzollini, Milena Mariani, Bernard Peissel, Siranoush Manoukian

**Affiliations:** Unit of Medical Genetics, Department of Medical Oncology and Hematology, Fondazione IRCCS Istituto Nazionale dei Tumori, Milan, Italy

Dear Editor,

We read with interest the recent article from O’Shea et al. in *Familial Cancer* [1]. The authors highlight the potential impact of multi-gene panel testing on the definition of broader phenotypes for cancer predisposing syndromes. Underlying this phenomenon is the increasing access to these next generation sequencing-based analyses, which are now frequently offered regardless of the clinical criteria used to select patients eligible for single-gene testing.

The *TP53* gene, mainly because of the wide range of associated cancers, represents a remarkable example of this process, being included in most commercially available multi-gene panels. Moreover, with the unrelenting increase of the breast cancer (BrCa) incidence in young women [2] and following the introduction of early-onset (<31 years) BrCa without significant family history in some of the current clinical guidelines, including the latest revision of the Chompret criteria [3], the number of *TP53* analyses is constantly increasing.

However, since the *TP53* genetic testing induces a certain degree of psychological distress, a more precise estimate of the a priori probability of harbouring a mutation would be helpful in the context of genetic counseling. A few studies on women affected with early-onset BrCa, who resulted negative at the *BRCA1*/*2* analysis and did not meet classic Li-Fraumeni (LFS) or Li-Fraumeni like (LFL) testing criteria, documented a mutation prevalence of 3–8% [3, 4], whereas others, including the study by O’Shea et al., did not detect any mutation at all [1, 5].

At the time of writing, at our Institution the *TP53* analysis has been performed in 197 patients, either from families fulfilling LFS/LFL criteria or BRCA-negative women affected with BrCa <31 years of age regardless of family history. The analysis was carried out through Sanger sequencing in all cases, in most cases genomic rearrangements were also investigated. Pathogenic mutations, classified according to the American College of Medical Genetics guidelines (ACMG) [6], were found in 8.6% (17/197) of probands, all of whom (5 males and 12 females) belong either to LFS (6) or LFL (11) families. Ten out of 12 women developed BrCa, with a median age at diagnosis of 30 years (range 20–55 years). As observed by O’Shea et al. in their cohort, also our low mutation detection rate (DR) may be explained by the inclusion of families with only cases of BrCa, since we mainly assess probands/families for hereditary breast cancer. When we considered only the 53 out of 197 probands affected with BrCa before 31 years of age, unselected for family history, the DR would be 9.4% (5/53), although noticeably each mutation carrier also showed a significant personal or family history (two classified as LFS, three as LFL) (Table 1).

**Table 1:** Characteristics of *TP53* mutation carriers affected with breast cancer before 31 years of age

Proband	*TP53* mutation	Breast cancer age	Breast cancer type^b^	Other cancers, age	Cancers in family members, age (degree of kinship)
1	c.844C>T (p.R282W)	20	IDC+ILC (G3, ER/PgR positive, HER2 positive)	–	Rhabdomyosarcoma, 3 (brother)
2	c.559G>A (p.V173fs59*)	28	DCIS (N.A.)	Sarcoma, 28	Gallbladder, 54 (mother) Breast, 55 (mother) Melanoma, 43 (2nd maternal) Melanoma 78 (2nd maternal)
3	c.309C>A (p.Y103*)	27	IDC (G3, ER/PgR positive, HER2 positive)	Osteosarcoma, 17	Breast, 31 (mother)
4	c.375+2T>G (p.?)	25, 30^a^	IDC (G3, ER positive, PgR negative, HER2 N.A.) IDC (G3, ER/PgR negative, HER2 negative)^a^	Rhabdomyosarcoma, 3 Osteosarcoma, 12	Glioblastoma, 19 (brother)
5	c.97-11C>G (p.?)	27, 27^a^	IDC (G3, ER/PgR negative, HER2 N.D.) DCIS (N.A.)^a^	Adrenocortical, 28	Chondrosarcoma, 46 (brother) Breast, 29 (mother) Breast, 36 (2nd maternal) Breast, 25, 27^a^ (3rd maternal) Central Nervous System, 10 (3rd maternal)

*IDC* invasive ductal carcinoma, *ILC* invasive lobular carcinoma, *DCIS* ductal carcinoma in situ, *ER* estrogen receptors, *PgR* progesterone receptors, *N.A*. not available

^a^Bilateral breast cancer

^b^Reported breast cancer features include histology, grade, hormone receptor and clinical HER2 status. “HER2 negative” includes immunohistochemistry scores 0, 1+ or 2+ (not amplified by in situ hybridization), “HER2 positive” includes immunohistochemistry scores 2+ (amplified by in situ hybridization) or 3+

These data elicit a few considerations concerning the *TP53* testing. Currently, the multi-gene testing panels for BrCa are widely used, both in clinics and research, and likely to include most of the *TP53* analyses performed. These tests will largely be offered to patients who do not meet classic LFS or LFL testing criteria, albeit some of whom will be affected with BrCa <31 years. The few studies reporting the *TP53* mutation prevalence in patients with early-onset BrCa are conflicting, some showing as in our case a very low DR in families not fulfilling LFS/LFL criteria, most of whom were also selected following a negative result at the *BRCA1*/*2* analysis. Conversely, other LFL criteria recently introduced, which do not consider other affected family members (e.g. childhood pathognomonic cancers or multiple primary tumours in the proband), seem to be more effective in identifying carriers, even in families with a de novo occurrence of a pathogenic mutation. Remarkably, despite the limited number of mutation carriers in our cohort, the LFL testing criteria allowed us to identify one de novo mutation in a girl, who was affected with rhabdomyosarcoma at the age of 3 and osteosarcoma at the age of 7.

It might be hypothesised that, since the genomic rearrangements analysis was not carried out in all of our patients, we might have missed some mutations, in particular in putative de novo cases such as isolated early-onset BrCa. Nevertheless, since the introduction as a testing criteria of BrCa <31 years irrespective of family history, we performed the deletions/duplications analysis in all probands. In addition, considering the low frequency of *TP53* genomic rearrangements, the limited number of patients in our cohort not examined with this assay would unlikely influence the global DR.

Another relevant aspect, which is rarely addressed, concerns patients who willingly decide not to undergo *TP53* testing. As we noticed a significant number of such cases, since December 2014 we have kept a track record of declined testing. Of note, 23.4% (11/47) of individuals, who were offered the *TP53* analysis following genetic counseling, declined to be tested. This proportion is exceptionally high, especially if compared with the declining of the *BRCA1*/*2* analysis within the same time span, occurring in <2% (13/776) of cases and with a steadily decreasing trend (3/352, <1% during the last year). A possible explanation of this phenomenon is that, though the use of genetic testing for preventive purposes has kept increasing in daily clinical practice along with testing acceptance, investigating a condition that lacks of effective preventive measures for all the expected cancers, such as LFS, still conveys a heavy psychological burden in a considerable proportion of individuals.

Genetic counseling and testing are now frequently requested shortly after diagnosis in order to inform treatment in women affected with BrCa. The identification of a mutation in high-penetrance genes, including *TP53*, might heavily influence the decisions about treatment procedures. Nevertheless, since December 2014, 13% (3/23) of our newly diagnosed patients, eligible to the *TP53* analysis, consented to undergo the *BRCA1*/*2* analysis but declined *TP53* testing. Based on these considerations it is thus to be acknowledged that the *TP53* analysis is a peculiar type of testing, which should be offered in the context of a thorough genetic counseling. In order to reduce the testing-related distress we now avoid, if feasible with respect to treatment timing, concurrent *BRCA1*/*2* and *TP53* analyses, offering the latter as a second-line testing even in newly diagnosed women who need to define treatment strategies.

Although, as underlined by O’Shea et al., multi-gene panels for BrCa are highly cost-effective, allow to reduce testing times and may improve our understanding of the phenotype of cancer-predisposing syndromes, they also make more challenging a comprehensive genetic counseling. Therefore, in order to avoid adverse effects correlated with unnecessary or unwanted testing, it seems appropriate to consider panel testing which include *TP53* in routine clinical practice with caution, making use of the clinical selection criteria as a valuable tool for advising genetic testing.
